# Abnormal brain white matter microstructure is associated with both pre-hypertension and hypertension

**DOI:** 10.1371/journal.pone.0187600

**Published:** 2017-11-16

**Authors:** Hideaki Suzuki, He Gao, Wenjia Bai, Evangelos Evangelou, Ben Glocker, Declan P. O’Regan, Paul Elliott, Paul M. Matthews

**Affiliations:** 1 Division of Brain Sciences, Department of Medicine, Hammersmith Hospital, Imperial College London, London, United Kingdom; 2 Department of Epidemiology and Biostatistics, Medical Research Council-Public Health England (MRC-PHE) Centre for Environment and Health, School of Public Health, Imperial College London, London, United Kingdom; 3 Biomedical Image Analysis Group, Department of Computing, Imperial College London, London, United Kingdom; 4 Medical Research Council London Institute of Medical Sciences, Hammersmith Hospital, Imperial College London, London, United Kingdom; University of Minnesota, UNITED STATES

## Abstract

**Objectives:**

To characterize effects of chronically elevated blood pressure on the brain, we tested for brain white matter microstructural differences associated with normotension, pre-hypertension and hypertension in recently available brain magnetic resonance imaging data from 4659 participants without known neurological or psychiatric disease (62.3±7.4 yrs, 47.0% male) in UK Biobank.

**Methods:**

For assessment of white matter microstructure, we used measures derived from neurite orientation dispersion and density imaging (NODDI) including the intracellular volume fraction (an estimate of neurite density) and isotropic volume fraction (an index of the relative extra-cellular water diffusion). To estimate differences associated specifically with blood pressure, we applied propensity score matching based on age, sex, educational level, body mass index, and history of smoking, diabetes mellitus and cardiovascular disease to perform separate contrasts of non-hypertensive (normotensive or pre-hypertensive, N = 2332) and hypertensive (N = 2337) individuals and of normotensive (N = 741) and pre-hypertensive (N = 1581) individuals (p<0.05 after Bonferroni correction).

**Results:**

The brain white matter intracellular volume fraction was significantly lower, and isotropic volume fraction was higher in hypertensive relative to non-hypertensive individuals (N = 1559, each). The white matter isotropic volume fraction also was higher in pre-hypertensive than in normotensive individuals (N = 694, each) in the right superior longitudinal fasciculus and the right superior thalamic radiation, where the lower intracellular volume fraction was observed in the hypertensives relative to the non-hypertensive group.

**Significance:**

Pathological processes associated with chronically elevated blood pressure are associated with imaging differences suggesting chronic alterations of white matter axonal structure that may affect cognitive functions even with pre-hypertension.

## Introduction

Hypertension affects as many as 1 billion people in the world [1] and damages multiple organ systems -including the brain- as a consequence of pathological changes affecting large and small vessels [2]. Hypertension and its secondary pathologies of stroke and small vessel disease are major risk factors for dementia [3]. Asymptomatic brain pathological changes found in people with chronic hypertension, such as lacunar infarction, white matter hyperintensities, and microbleeds, are associated independently with both early cognitive impairments and dementia [4–6]. The reduction in brain volume [7–9] and changes in white matter microstructure [10–16] that accompany these pathologies provide indirect evidence for neurodegeneration and oligodendroglial injury [17–19]. This neuronal and glial damage may be mediated by multiple mechanisms, including chronic or recurrent hypoxia-ischemia and inflammation [20–23].

Diffusion tensor imaging (DTI) has been used previously to evaluate hypertension-associated differences in white matter microstructure in efforts to assess the pathology in its earliest stages [10–16]. However, DTI-derived metrics including fractional anisotropy (FA), mean diffusivety (MD), and diffusion tensor mode (MO) lack specificity for individual tissue microstructure features [24]. A related, but improved, quantitative method, known as “neurite orientation dispersion and density imaging” (NODDI), uses a three-compartment tissue (including intracellular, extracellular and cerebrospinal fluid) model to increase sensitivity to differences in brain microstructure [25]. Parameters that can be derived from NODDI include the intracellular volume fraction (ICVF, an estimate of neurite density), orientation dispersion (OD, which expresses the extent of directional complexity of diffusion), and isotropic volume fraction (ISOVF, an index of the relative extra-cellular water diffusion) [25,26]. We believe that NODDI will allow a more sensitive and specific evaluation of white matter microstructure differences related to high blood pressure.

Here we describe white matter microstructure variations associated with normotension, pre-hypertension, unmedicated and medicated hypertension in an early release of data from 4939 UK Biobank (UKB) participants using measures derived from NODDI, as well as traditional DTI metrics. Pre-hypertension is a condition intermediated between normotension and hypertension [27]. Patients with pre-hypertension are encouraged to make lifestyle changes in order to delay or prevent progression to hypertension, but more active interventions have not been recommended to date [27]. However, the thresholds for effects of chronically elevated blood pressure on the brain have not been well defined.

We investigated first whether there were differences in white matter microstructure between non-hypertensive (normotensive and pre-hypertensive together) and hypertensive participants. Secondarily, we also tested whether the brain structural measures were also different between normotensive and pre-hypertensive participants and between unmedicated and medicated hypertensive participants.

## Materials and methods

### Study participants

UKB recruited about 500,000 community-dwelling participants aged 40–69 years across Great Britain between 2006 and 2010 [28,29]. Since 2014, a subset of participants underwent brain magnetic resonance imaging (MRI). Initially, data of 4939 individuals were available that included both brain volume and white matter microstructural MRI measures.

UKB received ethical approval from a nationally recognized research ethics committee (REC reference 11/NW/0382). The present study (the Biobank Brain and Cardiac Mutual Risk Indexing [BBC MRI] Study) was conducted under terms of UKB access approval 18545. Participants provided written informed consent as described on the UKB website [30]. If the participant who wished to take part was physically unable to provide a signature, two paper copies of the consent form were signed by two members of staff: the one witnessing consent and another, one of whom must be a qualified nurse [30].

### Baseline characteristics

Information on age, sex, educational level, body mass index (BMI), antihypertensive medication use, and history of smoking, diabetes mellitus, and cardiovascular disease were reported by participants at the time of the UKB imaging assessment.

Age was calculated by subtracting the birthdate of each participants from the imaging assessment date, which was recorded in the header of each image file. Educational level was treated as a binary variable indicating whether or not a participant achieved a college or university degree [31]. Information on reported cardiovascular disease and neurological or psychiatric disease were obtained separately from the ICD 10 codes in the UKB database.

### Blood pressure measurement

Details of the procedure for blood pressure measurement for UKB are available online [32]. Briefly, each participant was asked to sit and then an appropriate sized cuff was positioned on the upper arm, which was placed the arm on the desk top at the level of their heart. The right arm was used only if use of the left was not practical. After being asked to breathe in and out slowly five times in a relaxed fashion, the cuff on the blood pressure monitor (OMRON Healthcare Europe, NA, Hoofddorp) was inflated for the automated measure. A second measurement was conducted after at least 1 minute of rest. For this report, we used only blood pressure measurements acquired at the time of the imaging visit. We did not use those from the time of either the earlier UK Biobank initial assessment (2006–2010) or first repeat assessment visit (2012–2013). Systolic (SBP) and diastolic blood pressure (DBP) values were averaged over first and second measurements. We defined individuals as hypertensive if they had SBP ≥140 or DBP ≥90 mmHg or were receiving antihypertensive medication. Individuals with pre-hypertension were defined as having SBP of 120–139 mmHg or DBP of 80–89 mmHg. Normotensive individuals had SBP <120 or DBP <80 mmHg [27, 33].

### Brain MRI acquisition

Details of the image acquisition are available online [34]. Magnetic resonance imaging (MRI) was performed using a Siemens Skyra 3T running VD13A SP4 (Siemens Healthcare, Erlangen, Germany) with a Siemens 32-channel RF receive head coil. Diffusion MRI images were obtained using a Stejskal-Tanner pulse sequence with two b-values (b = 1000 and 2000 s/mm^2^), and a 2mm spatial resolution (3× multislice acquisition, 100 distinct diffusion-encoding directions, and a field-of-view of 104×104×72). T1-weighted structural brain images were obtained using a three-dimensional MPRAGE sequence with a slice thickness of 1mm and a field-of-view of 208×256×256.

### Brain MRI analyses

Details of the image processing for data provided from UKB dataset are available online and in the methods descriptions of previous reports [26, 31, 35]. The white matter microstructure (FA, MD, MO, ICVF, ISOVF and OD) in 27 anatomically defined white matter tracts described in the UKB dataset [26, 31] and brain volume measures (total brain volume (TBV), grey matter volume (GMV) and white matter volume (WMV)) were used. We derived participant summary indices for these white matter microstructure measures by averaging z scores for the 27 white matter tracts for each subject. Global values for each of the white matter microstructural measures are expressed as mean z-scores referenced to the mean measurement values for the total population studies (N = 4659). TBV, GMV and WMV all were normalized for intracranial volume.

**Fig 1:**
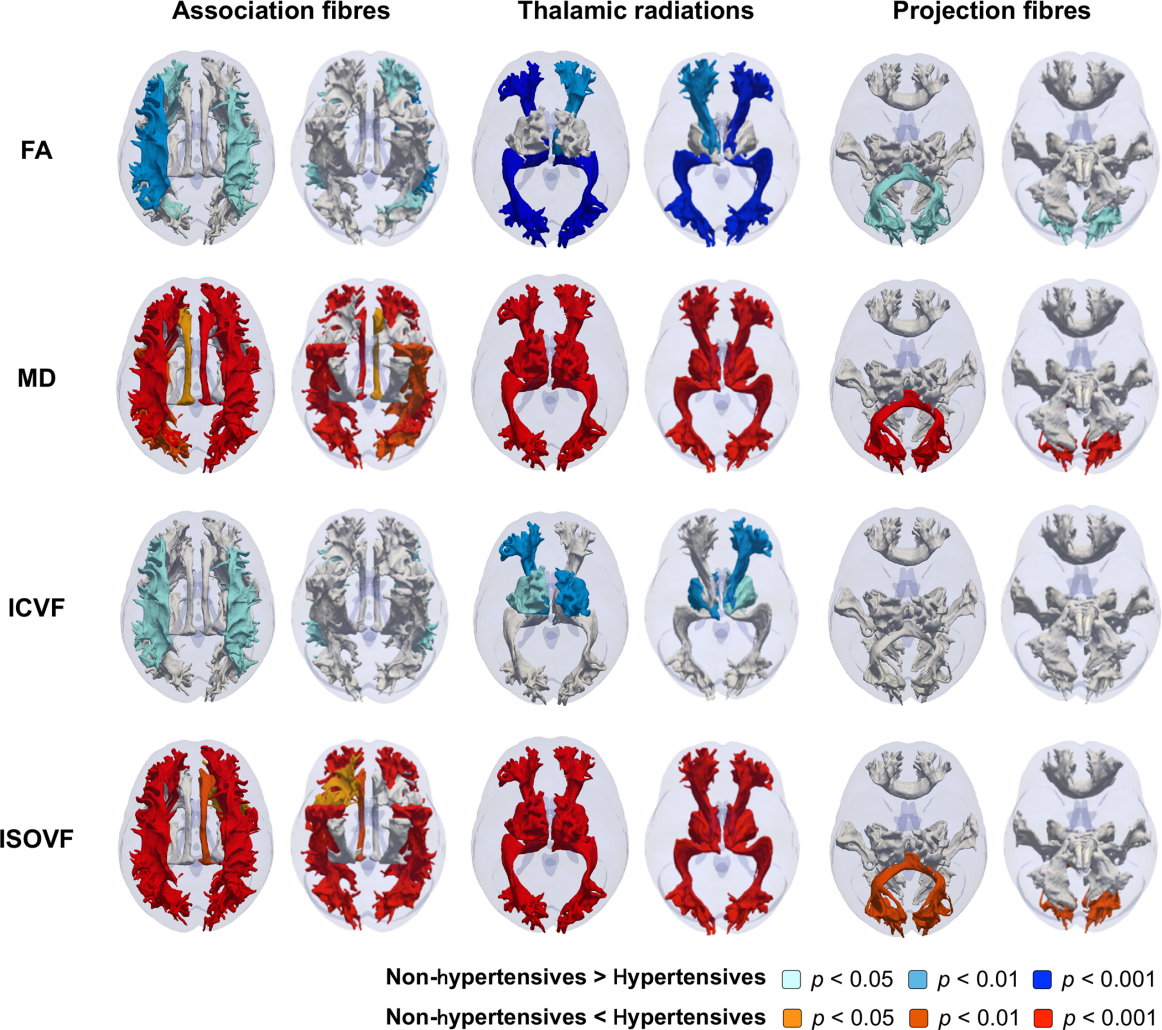
Illustrative representation of the contrast between microstructure measures of each 27 white matter tracts in the hypertensive and non-hypertensive groups. Probabilistic tractographies colored by *p* values adjusted for Bonferroni correction were shown from top and down. White-colored tracts indicate adjusted *p*>0.05. Abbreviations: FA, fractional anisotropy; ICVF, intracellular volume fraction; ISOVF, isotropic volume fraction; MD, mean diffusivity.

**Fig 2:**
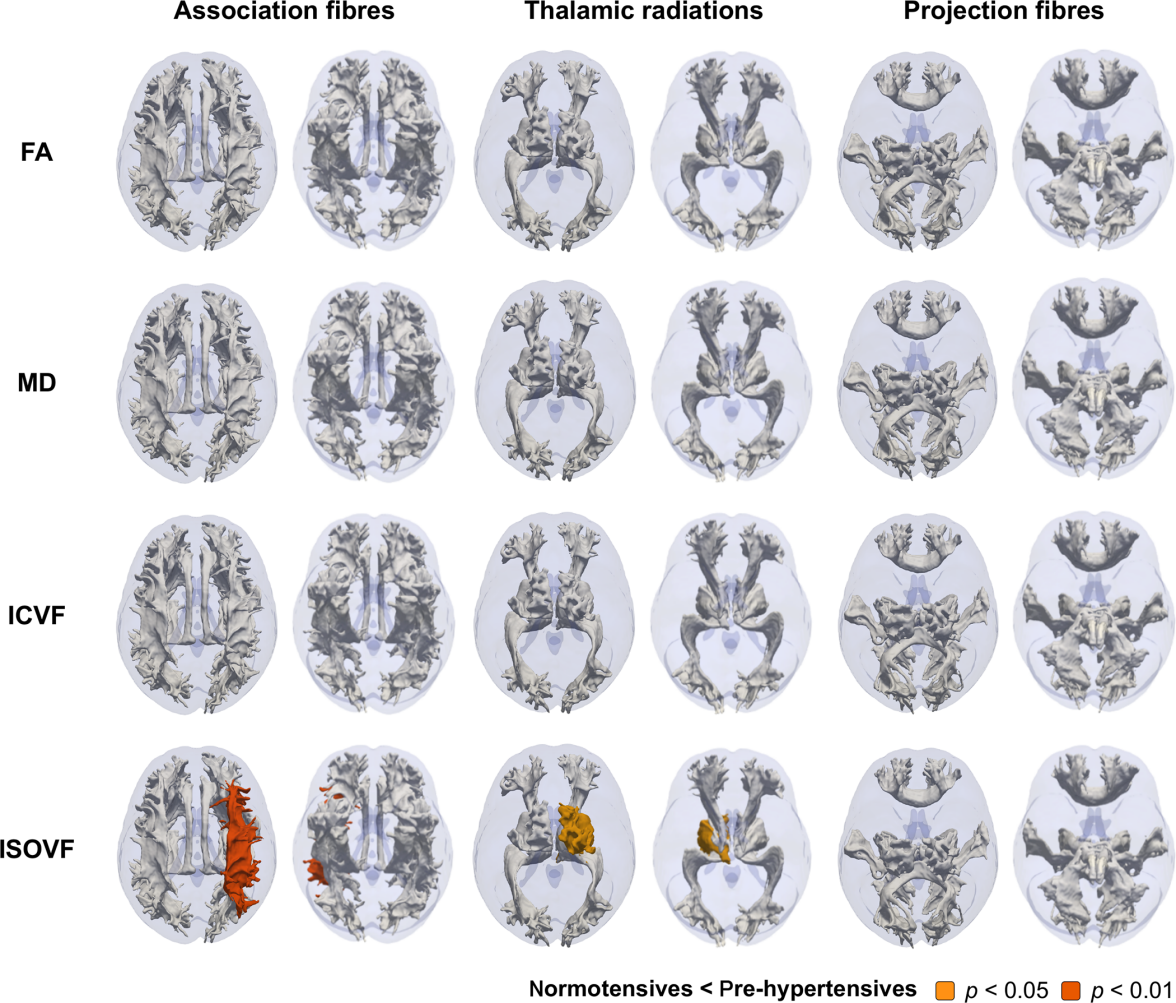
Illustrative representation of the contrast between microstructure measures of each 27 white matter tracts in the pre-hypertensive and normotensive groups. Probabilistic tractographies colored by *p* values adjusted for Bonferroni correction were shown from top and down. White-colored tracts indicate adjusted *p*>0.05. Abbreviations: FA, fractional anisotropy; ICVF, intracellular volume fraction; ISOVF, isotropic volume fraction; MD, mean diffusivity.

**Fig 3:**
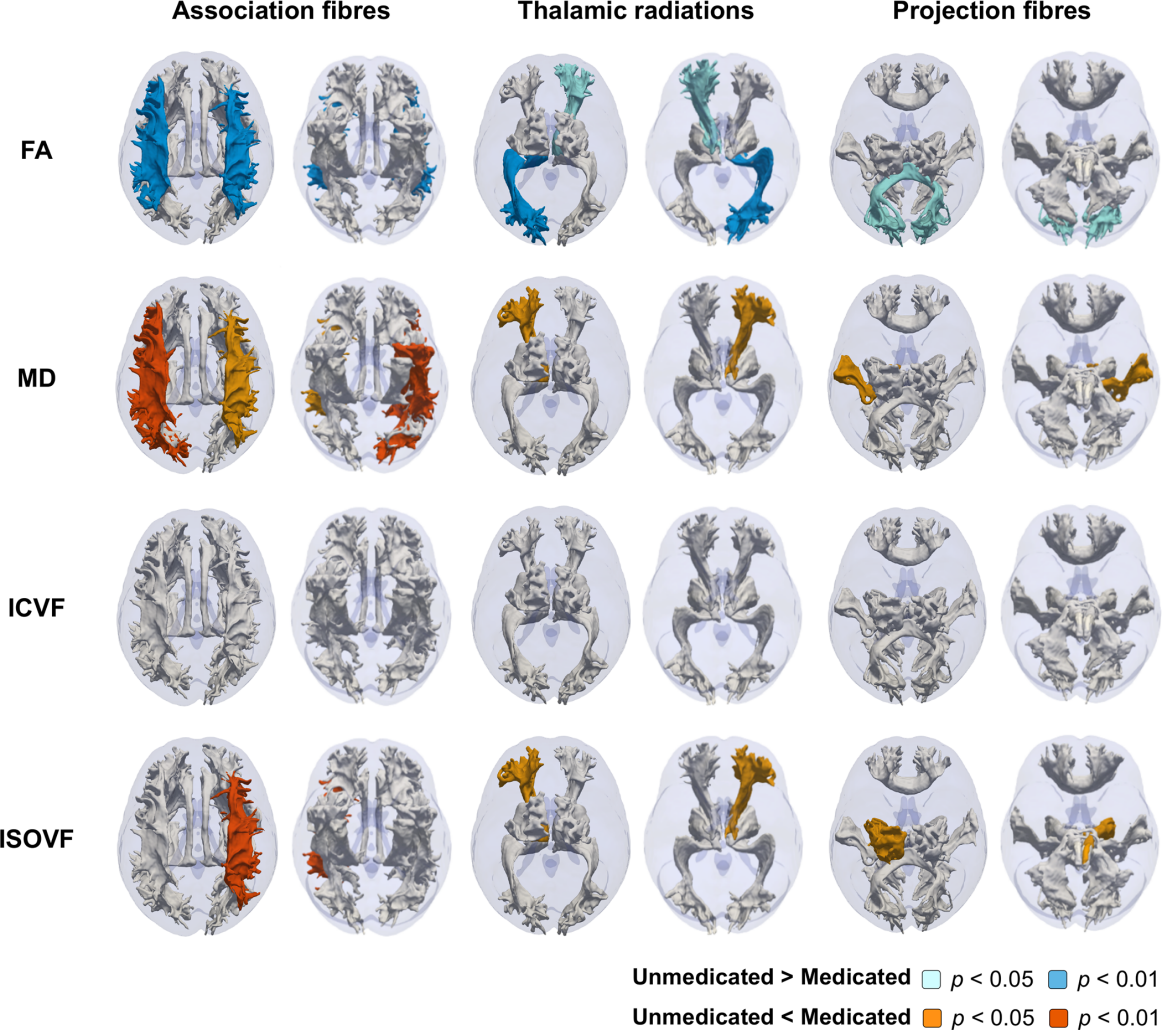
Illustrative representation of the contrast between microstructure measures of each 27 white matter tracts in the medicated and unmedicated groups. Probabilistic tractographies colored by *p* values adjusted for Bonferroni correction were shown from top and down. White-colored tracts indicate adjusted *p*>0.05. Abbreviations: FA, fractional anisotropy; ICVF, intracellular volume fraction; ISOVF, isotropic volume fraction; MD, mean diffusivity.

Illustrative, probabilistically, defined tract trajectories shown in **Figs 1–3**was made as described previously [36]. Briefly, surface meshes were made from probabilitstic descriptions of 27 white matter tracts in one UK Biobank participant. The tracts meshes were rendered as a 3D fashion onto a transparent brain mask that was derived from the MRI template available in SPM 12. Tract classifications as association fibres, thalamic radiations, and projection fibres were made based on assignments from a previous study [31].

### Statistical analysis

Continuous variables were analyzed using Student’s t-test and expressed as means ± standard deviations (non-hypertensives vs. hypertensives, normotensives vs. pre-hypertensive, or unmedicated vs medicated) except for the results of tract-based analyses, where probabilistic tractographies were colored by adjusted *p* values. Nominal variables were analyzed using Pearson’s chi-square statistic. Significance levels were set at *p*<0.05 adjusted after Bonferroni correction for multiple comparisons.

We classified individuals into normotensive (N = 741), pre-hypertensive (N = 1581), unmedicated (N = 1290) and medicated (N = 1047) hypertensive groups. We first conducted propensity score matching analysis to better balance groups for relevant variables other than hypertension. To obtain equal distributions of baseline characteristics between the non-hypertensive (normotensive and pre-hypertensive people, N = 2332) and hypertensive (unmedicated and medicated people, N = 2337) groups except for their blood pressure and antihypertensive medication, people in the non-hypertensive group were matched in a 1:1 ratio with the hypertensive group by baseline characteristics including age, sex, educational level, BMI, and history of smoking, DM and CVD through propensity score matching using the nearest neighbor method [37]. We then performed another propensity score matching for the baseline characteristics between the normotensive and pre-hypertensive groups and for the baseline characteristics and systolic and diastolic blood pressure between the unmedicated and medicated hypertensive groups, respectively. After propensity matching, we compared the groups for each of the brain structural measures. White matter microstructural measures were assessed globally and in 27 probabilistically defined white matter tracts.

## Results

### Participant characteristics

Of 4939 individuals who had both brain volume and white matter microstructural MRI measures, we excluded from this group those individuals lacking full baseline characterizations (N = 115), or two blood pressure measures (N = 62) and those with brain neurological/psychiatric disease (N = 103); 4659 individuals were analyzed in this study.

Before propensity matching, characteristics of people who were normotensive, pre-hypertensive, or unmedicated or medicated hypertensives showed differences in mean age, sex, educational level, BMI and history of DM and CVD (**Table 1**).

**Table 1:** Participant characteristics.

	Non-hypertensive (N = 2322)		Hypertensive (N = 2337)	
	Normotensive	Pre-hypertensive	Unmedicated	Medicated
N	741	1581	1290	1047
Baseline characteristics				
Age (yrs)	59.0±7.1	61.0±7.5	63.1±7.0	65.6±6.2
Male (%)	21.6	46.4	53.3	58.2
Educational level (%)	52.0	47.9	41.9	41.1
Body mass index	24.5±3.5	26.1±4.0	27.0±4.4	28.5±4.6
Ever smoked (%)	52.0	53.4	53.9	56.4
Diabetes mellitus (%)	1.8	2.5	3.0	11.7
Cardiovascular disease (%)	1.2	1.7	2.3	9.3
Blood pressures and antihypertensive medication				
Systolic blood pressure (mmHg)	111.8±6.4	129.6±5.8	152.3±11.8	142.6±17.0
Diastolic blood pressure (mmHg)	68.5±6.0	76.5±6.4	86.2±9.1	81.3±9.6
Antihypertensive medication (%)	0.0	0.0	0.0	100.0

Continuous variables were expressed as means ± standard deviations.

### Comparisons between non-hypertensive and hypertensive groups

To accurately estimate differences in brain structure between the non-hypertensive and hypertensive groups, we applied propensity score matching to control for confounding differences in baseline characteristics (**Table 2**). We tested for associations between hypertension and global white matter microstructure and brain volume measures (**Table 3**). While none of the brain volume measures were different between the matched groups, white matter FA (0.041±0.613 vs. –0.043±0.653, adjusted *p* = 0.001) and ICVF (0.036±0.788 vs. –0.042±0.836, adjusted *p* = 0.046) were significantly lower, and MD (–0.070±0.616 vs. 0.063±0.680, adjusted *p*<0.001) and ISOVF (–0.071±0.510 vs. 0.059±0.580, adjusted *p*<0.001) were higher among the hypertensives than in the non-hypertensive group.

**Table 2:** Participant characteristics in non-hypertensive and hypertensive groups after propensity score matching.

	Non-hypertensive	Hypertensive	p value
N	1559	1559	
Baseline characteristics			
Age (yrs)	62.6±7.0	62.6±6.8	0.815
Male (%)	48.0	47.7	0.858
Educational level (%)	45.5	44.6	0.614
Body mass index	26.5±4.0	26.5±3.9	0.826
Ever smoked (%)	53.8	54.1	0.886
Diabetes mellitus (%)	2.9	3.5	0.358
Cardiovascular disease (%)	2.2	2.6	0.559
Blood pressures and antihypertensive medication			
Systolic blood pressure (mmHg)	125.6±9.5	148.0±14.7	<0.001
Diastolic blood pressure (mmHg)	74.5±7.1	84.5±9.4	<0.001
Antihypertensive medication (%)	0.0	38.7	<0.001

The non-hypertensive group includes normotensive and pre-hypertensive individuals. Variables were expressed as means ± standard deviations and were analyzed using the Student’s t-test. Nominal variables were analyzed using the Pearson’s chi-square statistic.

**Table 3:** White matter microstructure measures in non-hypertensive and hypertensive groups after propensity score matching.

	Non-hypertensive	Hypertensive	p value
N	1559	1559	
White matter microstructure measure			
FA	0.041±0.613	–0.043±0.653	0.001
MD	–0.070±0.616	0.063±0.680	<0.001
MO	0.000±0.341	–0.006±0.353	1.000
ICVF	0.036±0.788	–0.042±0.836	0.046
OD	0.013±0.483	–0.014±0.497	0.706
ISOVF	–0.071±0.510	0.059±0.580	<0.001
Brain volume measure			
Total brain volume (ml)	1506.4±71.0	1506.7±70.9	1.000
Grey matter volume (ml)	796.8±45.8	795.8±44.4	1.000
White matter volume (ml)	709.6±40.7	710.8±41.5	1.000

Variables were expressed as means ± standard deviations and were analyzed using the Student’s t-test. White matter microstructural measures are expressed as mean z-scores referenced to the total study population (n = 4659). Significance levels were set at p<0.05 after Bonferroni correction. Abbreviations: FA, Fractional anisotropy; ICVF, Intracellular volume fraction; ISOVF, Isotropic volume fraction; MD, Mean diffusivity; MO, Diffusion tensor mode; OD, Orientation dispersion.

We next tested for hypertensive group differences in microstructure of 27 white matter tracts (**Fig 1**). Both higher MD and higher ISOVF were found in the hypertensive group in the bilateral inferior fronto-occipital fasciculus, the right cingulate gyrus part of cingulum, the bilateral superior and inferior longitudinal fasciculus, the bilateral three thalamic radiations, and the forceps minor. The cingulum bundle and the right uncinate fasciculus showed only higher MD or higher ISOVF, respectively (adjusted p<0.05, both). Greater FA in the hypertensive group was found in the left inferior fronto-occipital fasciculus, the bilateral superior and right inferior longitudinal fasciculus, the bilateral anterior and posterior thalamic radiation, and the forceps minor (adjusted p<0.05, all). The bilateral superior longitudinal fasciculus and the left anterior and bilateral superior thalamic radiations showed greater ICVF (adjusted p<0.05, all).

### Comparisons between normotensive and pre-hypertensive groups

We then performed propensity score matching between the normotensive and pre-hypertensive groups to test whether a lower blood pressure threshold distinguished structural brain differences (**Table 4**). While the results of the other white matter microstructure and brain volume measures were not significant, an association of higher ISOVF again was found in the global measure (–0.178±0.479 vs. –0.097±0.517, *adjusted p* = 0.015) (**Table 5**) and, more specifically, in the right superior longitudinal fasciculus and the right superior thalamic radiation (adjusted *p*<0.05) (**Fig 2**).

**Table 4:** Participant characteristics in normotensive and pre-hypertensive groups after propensity score matching.

	Normotensive	Pre-hypertensive	p value
N	694	694	
Baseline characteristics			
Age (yrs)	59.3±7.1	59.5±7.2	0.724
Male (%)	23.1	23.9	0.704
Educational level (%)	50.7	48.7	0.452
Body mass index	24.7±3.4	24.6±3.4	0.507
Ever smoked (%)	52.2	53.6	0.591
Diabetes mellitus (%)	1.9	1.2	0.272
Cardiovascular disease (%)	1.2	0.9	0.591
Blood pressures and antihypertensive medication			
Systolic blood pressure (mmHg)	111.9±6.3	129.0±5.8	<0.001
Diastolic blood pressure (mmHg)	68.6±5.9	76.2±6.4	<0.001

The non-hypertensive group includes normotensive and pre-hypertensive individuals. Variables were expressed as means ± standard deviations and were analyzed using the Student’s t-test. Nominal variables were analyzed using the Pearson’s chi-square statistic.

**Table 5:** White matter microstructure measures in normotensive and pre-hypertensive groups after propensity score matching.

	Non-hypertensive	Hypertensive	p value
N	694	694	
White matter microstructure measure			
FA	0.048±0.586	0.029±0.604	1.000
MD	–0.171±0.579	–0.127±0.591	0.949
MO	–0.033±0.337	–0.033±0.331	1.000
ICVF	0.066±0.751	0.072±0.762	1.000
OD	0.093±0.473	0.103±0.495	1.000
ISOVF	–0.178±0.479	–0.097±0.517	0.015
Brain volume measure			
Total brain volume (ml)	1531.2±72.4	1526.9±71.6	0.774
Grey matter volume (ml)	817.5±45.3	815.8±45.7	1.000
White matter volume (ml)	713.8±40.5	711.1±39.9	0.629

Variables were expressed as means ± standard deviations and were analyzed using the Student’s t-test. White matter microstructural measures are expressed as mean z-scores referenced to the total study population (n = 4659). Significance levels were set at p<0.05 after Bonferroni correction. Abbreviations: FA, Fractional anisotropy; ICVF, Intracellular volume fraction; ISOVF, Isotropic volume fraction; MD, Mean diffusivity; MO, Diffusion tensor mode; OD, Orientation dispersion.

### Comparisons between unmedicated and medicated hypertensive groups

We also tested for any difference in brain imaging measures in people being treated with anti-hypertensive medications relative to those who were hypertensive and not receiving specific medication, adjusting for baseline characteristics and blood pressure (**Table 6**). Global white matter microstructure measures showed lower FA (0.028±0.619 vs. –0.102±0.691, adjusted *p* = 0.002) and higher MD (0.052±0.651 vs. 0.191±0.708, adjusted *p* = 0.001) and ISOVF (0.059±0.550 vs. 0.170±0.595, adjusted *p* = 0.002) in the medicated than in the unmedicated group (**Table 7**). Using tract-based analysis for this contrast showed lower FA in the bilateral superior longitudinal fasciculus, the right anterior and left posterior thalamic radiation, and the forceps minor, higher MD in the bilateral superior and left inferior longitudinal fasciculus, the left anterior thalamic radiation, and the left acoustic radiation, and higher ISOVF in the left superior longitudinal fasciculus, the left anterior thalamic radiation, and the left corticospinal tract (adjusted *p*<0.05, all) (**Fig 3**).

**Table 6:** Characteristics of unmedicated and medicated hypertensive participants after propensity score matching.

	Unmedicated	Medicated	p value
N	672	672	
Baseline characteristics			
Age (yrs)	64.7±6.9	64.6±6.3	0.817
Male (%)	55.7	55.1	0.587
Educational level (%)	43.3	42.9	0.869
Body mass index	27.6±4.7	27.6±4.2	0.784
Ever smoked (%)	54.9	54.6	0.913
Diabetes mellitus (%)	5.5	4.3	0.313
Cardiovascular disease (%)	3.7	3.1	0.548
Blood pressures and antihypertensive medication			
Systolic blood pressure (mmHg)	149.6±10.3	148.8±15.7	0.292
Diastolic blood pressure (mmHg)	84.3±9.1	84.1±9.3	0.714
Antihypertensive medication (%)	0	100.0	<0.001

Variables were expressed as means ± standard deviations and were analyzed using the Student’s t-test. Nominal variables were analyzed using the Pearson’s chi-square statistic.

**Table 7:** White matter microstructure measures in unmedicated and medicated participants with hypertension after propensity score matching.

	Unmedicated	Medicated	p value
N	834	834	
White matter microstructure measure			
FA	0.028±0.619	–0.102±0.691	0.002
MD	0.052±0.651	0.191±0.708	0.001
MO	0.013±0.352	–0.003±0.344	1.000
ICVF	–0.008±0.826	–0.116±0.875	0.123
OD	–0.059±0.501	–0.055±0.507	1.000
ISOVF	0.059±0.550	0.170±0.595	0.002
Brain volume measure			
Total brain volume (ml)	1496.2±68.8	1492.1±69.2	0.806
Grey matter volume (ml)	788.8±43.8	784.8±43.6	0.267
White matter volume (ml)	707.4±40.2	707.3±41.3	1.000

Variables were expressed as means ± standard deviations and were analyzed using the Student’s t-test. White matter microstructural measures are expressed as mean z-scores referenced to the total study population (n = 4659). Significance levels were set at p<0.05 after Bonferroni correction. Abbreviations: FA, Fractional anisotropy; ICVF, Intracellular volume fraction; ISOVF, Isotropic volume fraction; MD, Mean diffusivity; MO, Diffusion tensor mode; OD, Orientation dispersion.

## Discussion

Here we describe differences in white matter microstructure between non-hypertensive and hypertensive, normotensive and pre-hypertensive, and unmedicated and medicated hypertensive UKB participants. We found differences in FA, MD, ICVF and ISOVF between the non-hypertensive and hypertensive groups even after adjustment of possible confounding factors using propensity score matching. An association of higher ISOVF with higher blood pressure also was found in the contrast between normotensive and pre-hypertensive groups in the right superior longitudinal fasciculus and the right superior thalamic radiation, where the lower ICVF was observed in the hypertensives relative to the non-hypertensive group. These microstructural differences may contribute to future risks for cognitive impairment and dementia in the pre-hypertensive group. The comparison between unmedicated and medicated hypertensive participants unexpectedly showed lower FA and higher MD and ISOVF in the group treated with anti-hypertensive medication. A possible explanation for this is that medicated hypertensives had experienced more sustained, more severe hypertension, which had progressed irreversible abnormalities in white matter microstructure, before starting antihypertensive medication. Future work is needed to test directly whether white matter changes associated with hypertension are reversible after the effective treatment.

Our findings of lower white matter FA and higher MD in the hypertensives relative to the non-hypertensive group in this study are consistent with results of the previous studies [10–16]. Higher SBP previously was associated with lower FA within white matter tracts including the anterior corpus callosum, inferior fronto-occipital fasciculi, superior lateral fasciculi, uncinate and/or fibres that project from the thalamus to the superior frontal gyrus [13,14]. Mean arterial blood pressure was linearly correlated with FA in the whole white matter, corpus callosum, genu, parietal white matter, and superior and lateral frontal white matter [11,12]. Animal studies have validated these changes in diffusion MRI measures in terms of histopathological measures of white matter microstructure in other contexts [18,19]. Our study added evidence for differences in lower white matter ICVF and higher ISOVF in the hypertensive compared to the non-hypertensive group. They can be interpreted more specifically: ICVF provides an estimate of neurite density and ISOVF is a measure of extra-cellular water diffusion (ISOVF) [25,26]. Our results suggest that hypertension is associated with a lower density or packing of axons in the white matter.

A lower white matter ISOVF was observed even in pre-hypertensive participants in UKB that included our study. This indicates ISOVF appear more sensitive to brain pathology associated with chronically elevated blood pressure than are conventional diffusion MRI measures. Maillard et al. showed lower FA and higher MD in the anterior corpus callosum and inferior fronto-occipital fascicule and lower GMV in Brodmann’s area 21 and 48 in pre-hypertensives relative to a normotensive group [14]. Our study extends their finding by showing that ISOVF was lower in a pre-hypertensive group relative to normotensives even after controlling for potential confounds of age, sex, educational level, BMI, and history of smoking, DM and CVD.

The results of tract-based analyses provided a more detailed characterization of differences in white matter integrity associated with high blood pressure. Lower ICVF was observed in the superior longitudinal fasciculus and the superior thalamic radiation in hypertensives relative to non-hypertensives and ISOVF was higher in the same tracts in pre-hypertensives than in the normotensive group. This suggests both that these tracts are amongst the most vulnerable to changes in microstructure with high blood pressure and (to the extent that inference regarding possible longitudinal changes is possible from these cross-sectional observations) that changes in white matter ISOVF may precede those in ICVF with high blood pressure. This loss of white matter integrity being measured may have clinical significance, e.g., lower FA in the superior longitudinal fasciculus is associated with cognitive decline in hypertensive patients [38].

The lower ICVF indicates that hypertension may be associated with white matter dystrophy or axonal loss. The higher ISOVF associated with high blood pressure suggests increased extra-cellular water diffusion, in other pathologies, has been associated with increased blood-brain permeability or local inflammatory activation. For example, NODDI shows lower ICVF and higher ISOVF in white matter lesions of multiple sclerosis, which are characterized histopathologically by inflammatory demyelination associated with breakdown of the blood-brain barrier and axonal injury [39,40]. Hypertension also increases blood-brain permeability by affecting perivascular lymphatic drainage, resulting in the local accumulation of blood proteins that cause a toxic inflammatory response [21]. Even in the absence of evidence for frank blood-brain barrier permeability changes, vascular adaptation to chronic hypertension are associated with pro-inflammatory immune activation and tissue injury [22,23]. Independent associations of similar white matter pathology with cognitive impairment and a future risk of dementia suggest that they are not benign [41,42]. The potential impact of early antihypertensive medication on the trajectory of white matter microstructure damage needs further study.

Our observation of brain white matter differences in the pre-hypertensive group suggests that the optimal blood pressure for brain health may be lower than previously considered [14]. However, multiple factors should be taken into account. Controlling blood pressure to 130/80 mmHg reduced incidence of strokes in hypertensive patients with high cardiovascular risk [43]. However, longitudinal studies of populations of older people have shown associations of lower blood pressure with higher incidence of dementia [44]. A randomized control trial of intensive blood pressure therapy targeting SBP<120 mmHg accelerated the decrease in TBV with aging compared with standard therapy targeting SBP<140 mmHg in patients with type 2 DM [9]. The relationships between measures of white matter structural changes and clinical outcomes in these different contexts needs further study.

There are several limitations of this study. First, our observation focused on a younger population (mean age 62.3±7.4 yrs) with few co-morbid diseases. The future availability of a much larger population from the UKB should allow characterization of possible differences in association across well-defined subgroups, e.g., those at high cardiovascular risk and older subjects. Second, propensity score matching might choose participants who were less representative in original groups. Individuals with DM and/or CVD were more forced to make a match than those without comorbidities, who were more common. The results should be interpreted under a recognition of such an analysis-derived limitation. Third, this study was cross-sectional and observational. Study of relationships between these white matter changes and future cognitive impairment, stroke and dementia will become possible over time with the longitudinal data collection in UKB. However, a properly randomized, prospective, placebo controlled trial of antihypertensive therapy is needed to provide a confident basis for changes in current medical practice.

## Conclusions

Our study demonstrated that hypertension is associated with differences in white matter ICVF and ISOVF, suggesting axonal loss or dystrophy. An association of lower ISOVF also was associated with pre-hypertension. The superior longitudinal fasciculus and the superior thalamic radiation might be the most susceptible for high blood pressure. Our results suggest that small vessel pathological processes with chronically elevated blood pressure may lead to axonal pathology that can disrupt brain connectivity and contribute to impairment of cognitive functions even with pre-hypertension.

## References

[1] NCD Risk Factor Collaboration (NCD-RisC); Worldwide trends in blood pressure from 1975 to 2015: a pooled analysis of 1479 population-based measurement studies with 19·1 million participants. Lancet. 2017; 389: 37–55. doi: 10.1016/S0140-6736(16)31919-5 27863813 10.1016/S0140-6736(16)31919-5 PMC5220163

[2] Veglio F, Paglieri C, Rabbia F, Bisbocci D, Bergui M, Cerrato P. Hypertension and cerebrovascular damage. Atherosclerosis. 2009; 205: 331–341. doi: 10.1016/j.atherosclerosis.2008.10.028 19100549 10.1016/j.atherosclerosis.2008.10.028

[3] Iadecola C. Best papers in hypertension: Hypertension and dementia. Hypertension. 2014; 64: 3–5. doi: 10.1161/HYPERTENSIONAHA.114.03040 24777976 10.1161/HYPERTENSIONAHA.114.03040 PMC4234111

[4] Makin SD, Turpin S, Dennis MS, Wardlaw JM. Cognitive impairment after lacunar stroke: systematic review and meta-analysis of incidence, prevalence and comparison with other stroke subtypes. J Neurol Neurosurg Psychiatry. 2013; 84: 893–900. doi: 10.1136/jnnp-2012-303645 23457225 10.1136/jnnp-2012-303645 PMC3717603

[5] Prins ND, Scheltens P. White matter hyperintensities, cognitive impairment and dementia: an update. Nat Rev Neurol. 2015; 11: 157–165. doi: 10.1038/nrneurol.2015.10 25686760 10.1038/nrneurol.2015.10

[6] Miwa K, Tanaka M, Okazaki S, Yagita Y, Sakaguchi M, Mochizuki H, et al Multiple or mixed cerebral microbleeds and dementia in patients with vascular risk factors. Neurology. 2014; 83: 646–653. doi: 10.1212/WNL.0000000000000692 25015364 10.1212/WNL.0000000000000692

[7] Beauchet O, Celle S, Roche F, Bartha R, Montero-Odasso M, Allali G, et al Blood pressure levels and brain volume reduction: a systematic review and meta-analysis. J Hypertens. 2013; 31: 1502–1516. doi: 10.1097/HJH.0b013e32836184b5 23811995 10.1097/HJH.0b013e32836184b5

[8] Jochemsen HM, Muller M, Visseren FL, Scheltens P, Vincken KL, Mali WP, et al; SMART Study Group. Blood pressure and progression of brain atrophy: the SMART-MR Study. JAMA Neurol. 2013; 70: 1046–1053. doi: 10.1001/jamaneurol.2013.217 23753860 10.1001/jamaneurol.2013.217

[9] Williamson JD, Launer LJ, Bryan RN, Coker LH, Lazar RM, Gerstein HC, et al; Action to Control Cardiovascular Risk in Diabetes Memory in Diabetes Investigators. Cognitive function and brain structure in persons with type 2 diabetes mellitus after intensive lowering of blood pressure and lipid levels: a randomized clinical trial. JAMA Intern Med. 2014; 174: 324–333. doi: 10.1001/jamainternmed.2013.13656 24493100 10.1001/jamainternmed.2013.13656 PMC4423790

[10] Kennedy KM, Raz N. Pattern of normal age-related regional differences in white matter microstructure is modified by vascular risk. Brain Res. 2009; 1297: 41–56. doi: 10.1016/j.brainres.2009.08.058 19712671 10.1016/j.brainres.2009.08.058 PMC2758325

[11] Leritz EC, Salat DH, Milberg WP, Williams VJ, Chapman CE, Grande LJ, et al Variation in blood pressure is associated with white matter microstructure but not cognition in African Americans. Neuropsychology. 2010; 24: 199–208. doi: 10.1037/a0018108 20230114 10.1037/a0018108 PMC2849283

[12] Gons RA, van Oudheusden LJ, de Laat KF, van Norden AG, van Uden IW, Norris DG, et al Hypertension is related to the microstructure of the corpus callosum: the RUN DMC study. J Alzheimers Dis. 2012; 32: 623–631. doi: 10.3233/JAD-2012-121006 22869466 10.3233/JAD-2012-121006

[13] Salat DH, Williams VJ, Leritz EC, Schnyer DM, Rudolph JL, Lipsitz LA, et al Inter-individual variation in 39 blood pressure is associated with regional white matter integrity in generally healthy older adults. Neuroimage. 2012; 59: 181–192. doi: 10.1016/j.neuroimage.2011.07.033 21820060 10.1016/j.neuroimage.2011.07.033 PMC3209709

[14] Maillard P, Seshadri S, Beiser A, Himali JJ, Au R, Fletcher E, et al Effects of systolic blood pressure on white-matter integrity in young adults in the Framingham Heart Study: a cross-sectional study. Lancet Neurol. 2012; 11: 1039–1047. doi: 10.1016/S1474-4422(12)70241-7 23122892 10.1016/S1474-4422(12)70241-7 PMC3510663

[15] Rosano C, Abebe KZ, Aizenstein HJ, Boudreau R, Jennings JR, Venkatraman V, et al; Health ABC Study. Longitudinal systolic blood pressure characteristics and integrity of white matter tracts in a cohort of very old black and white adults. Am J Hypertens. 2015; 28: 326–334. doi: 10.1093/ajh/hpu134 25159083 10.1093/ajh/hpu134 PMC4325666

[16] McEvoy LK, Fennema-Notestine C, Eyler LT, Franz CE, Hagler DJ Jr, Lyons MJ, et al Hypertension-related alterations in white matter microstructure detectable in middle age. Hypertension. 2015; 66: 317–323. doi: 10.1161/HYPERTENSIONAHA.115.05336 26056337 10.1161/HYPERTENSIONAHA.115.05336 PMC4499000

[17] Suzuki H1, Sumiyoshi A, Taki Y, Matsumoto Y, Fukumoto Y, Kawashima R, et al Voxel-based morphometry and histological analysis for evaluating hippocampal damage in a rat model of cardiopulmonary resuscitation. Neuroimage. 2013; 77: 215–221. doi: 10.1016/j.neuroimage.2013.03.042 23558096 10.1016/j.neuroimage.2013.03.042

[18] Sampaio-Baptista C, Khrapitchev AA, Foxley S, Schlagheck T, Scholz J, Jbabdi S, et al Motor skill learning induces changes in white matter microstructure and myelination. J Neurosci. 2013; 33: 19499–19503. doi: 10.1523/JNEUROSCI.3048-13.2013 24336716 10.1523/JNEUROSCI.3048-13.2013 PMC3858622

[19] Calabrese E, Johnson GA. Diffusion tensor magnetic resonance histology reveals microstructural changes in the developing rat brain. Neuroimage. 2013; 79: 329–339. doi: 10.1016/j.neuroimage.2013.04.101 23648962 10.1016/j.neuroimage.2013.04.101 PMC3690820

[20] Moody DM, Bell MA, Challa VR. Features of the cerebral vascular pattern that predict vulnerability to perfusion or oxygenation deficiency: an anatomic study. AJNR Am J Neuroradiol. 1990; 11: 431–439. 2112304 PMC8367475

[21] Mohammadi MT, Dehghani GA. Acute hypertension induces brain injury and blood-brain barrier disruption through reduction of claudins mRNA expression in rat. Pathol Res Pract. 2014; 210: 985–990. doi: 10.1016/j.prp.2014.05.007 24996562 10.1016/j.prp.2014.05.007

[22] Rosenberg GA. Inflammation and white matter damage in vascular cognitive impairment. Stroke. 2009; 40: S20–S23. doi: 10.1161/STROKEAHA.108.533133 19064797 10.1161/STROKEAHA.108.533133 PMC2811584

[23] Harrison DG, Guzik TJ, Lob HE, Madhur MS, Marvar PJ, Thabet SR, et al Inflammation, immunity, and hypertension. Hypertension. 2011; 57: 132–140. doi: 10.1161/HYPERTENSIONAHA.110.163576 21149826 10.1161/HYPERTENSIONAHA.110.163576 PMC3028593

[24] Pierpaoli C, Jezzard P, Basser PJ, Barnett A, Di Chiro G. Diffusion tensor MR imaging of the human brain. Radiology. 1996; 201: 637–648. doi: 10.1148/radiology.201.3.8939209 8939209 10.1148/radiology.201.3.8939209

[25] Zhang H, Schneider T, Wheeler-Kingshott CA, Alexander DC. NODDI: practical in vivo neurite orientation dispersion and density imaging of the human brain. Neuroimage. 2012; 61: 1000–1016. doi: 10.1016/j.neuroimage.2012.03.072 22484410 10.1016/j.neuroimage.2012.03.072

[26] UK Biobank primary brain image documentation. Available: http://biobank.ctsu.ox.ac.uk/crystal/refer.cgi?id=1977

[27] Weber MA, Schiffrin EL, White WB, Mann S, Lindholm LH, Kenerson JG, et al Clinical practice guidelines for the management of hypertension in the community a statement by the American Society of Hypertension and the International Society of Hypertension. J Hypertens. 2014; 32: 3–15. doi: 10.1097/HJH.0000000000000065 24270181 10.1097/HJH.0000000000000065

[28] UK Biobank top page. Available: http://www.ukbiobank.ac.uk

[29] Sudlow C, Gallacher J, Allen N, Beral V, Burton P, Danesh J, et al UK biobank: an open access resource for identifying the causes of a wide range of complex diseases of middle and old age. PLoS Med. 2015; 12: e1001779 doi: 10.1371/journal.pmed.1001779 25826379 10.1371/journal.pmed.1001779 PMC4380465

[30] Date of consenting to join UK Biobank. Available: http://biobank.ctsu.ox.ac.uk/crystal/field.cgi?id=200

[31] Cox SR, Ritchie SJ, Tucker-Drob EM, Liewald DC, Hagenaars SP, Davies G, et al Ageing and brain white matter structure in 3513 UK Biobank participants. Nat commun. 2016; 7: 13629 doi: 10.1038/ncomms13629 27976682 10.1038/ncomms13629 PMC5172385

[32] UK Biobank blood-pressure measurement procedures. Available: http://biobank.ctsu.ox.ac.uk/crystal/refer.cgi?id=100225

[33] Verhaaren BF, Vernooij MW, de Boer R, Hofman A, Niessen WJ, van der Lugt A, et al High blood pressure and cerebral white matter lesion progression in the general population. Hypertension. 2013; 61:1354–1359. doi: 10.1161/HYPERTENSIONAHA.111.00430 23529163 10.1161/HYPERTENSIONAHA.111.00430

[34] UK Biobank brain scan protocol. Available: http://biobank.ctsu.ox.ac.uk/crystal/refer.cgi?id=2367

[35] Miller KL, Alfaro-Almagro F, Bangerter NK, Thomas DL, Yacoub E, Xu J, et al Multimodal population brain imaging in the UK Biobank prospective epidemiological study. Nat Neurosci. 2016; 19: 1523–1536. doi: 10.1038/nn.4393 27643430 10.1038/nn.4393 PMC5086094

[36] Madan CR. Creating 3D visualizations of MRI data: A brief guide. F1000Res. 2015; 4: 466 doi: 10.12688/f1000research.6838.1 26594340 10.12688/f1000research.6838.1 PMC4648228

[37] Austin PC. An Introduction to Propensity Score Methods for Reducing the Effects of Confounding in Observational Studies. Multivariate Behav Res. 2011; 46: 399–424. doi: 10.1080/00273171.2011.568786 21818162 10.1080/00273171.2011.568786 PMC3144483

[38] Balk LJ, Cruz-Herranz A, Albrecht P, Arnow S, Gelfand JM, Tewarie P, et al Timing of retinal neuronal and axonal loss in MS: a longitudinal OCT study. J Neurol. 2016; 263: 1323–1331. doi: 10.1007/s00415-016-8127-y 27142714 10.1007/s00415-016-8127-y PMC4929170

[39] Mallik S, Samson RS, Wheeler-Kingshott CA, Miller DH. Imaging outcomes for trials of remyelinationin multiple sclerosis. J Neurol Neurosurg Psychiatry. 2014; 85: 1396–1404. doi: 10.1136/jnnp-2014-307650 24769473 10.1136/jnnp-2014-307650 PMC4335693

[40] Schneider T, Brownlee W, Zhang H, Ciccarelli O, Miller DH, Wheeler-Kingshott CG. Sensitivity of multi-shell NODDI to multiple sclerosis white matter changes: a pilot study. Funct Neurol. 2017; 32: 97–101. doi: 10.11138/FNeur/2017.32.2.097 28676143 10.11138/FNeur/2017.32.2.097 PMC5507159

[41] Douaud G, Menke RA, Gass A, Monsch AU, Rao A, Whitcher B, et al Brain microstructure reveals early abnormalities more than two years prior to clinical progression from mild cognitive impairment to Alzheimer's disease. J Neurosci. 2013; 33: 2147–2155. doi: 10.1523/JNEUROSCI.4437-12.2013 23365250 10.1523/JNEUROSCI.4437-12.2013 PMC6571077

[42] Grieve SM, Williams LM, Paul RH, Clark CR, Gordon E. Cognitive aging, executive function, and fractional anisotropy: a diffusion tensor MR imaging study. AJNR Am J Neuroradiol. 2007; 28: 226–235. 17296985 PMC7977408

[43] Mancia G, Kjeldsen SE, Zappe DH, Holzhauer B, Hua TA, Zanchetti A, et al Cardiovascular outcomes at different on-treatment blood pressures in the hypertensive patients of the VALUE trial. Eur Heart J. 2016;37: 955–964. doi: 10.1093/eurheartj/ehv633 26590384 10.1093/eurheartj/ehv633

[44] Iadecola C, Yaffe K, Biller J, Bratzke LC, Faraci FM, Gorelick PB, et al; American Heart Association Council on Hypertension; Council on Clinical Cardiology; Council on Cardiovascular Disease in the Young; Council on Cardiovascular and Stroke Nursing; Council on Quality of Care and Outcomes Research; and Stroke Council. Impact of Hypertension on Cognitive Function A Scientific Statement From the American Heart Association. Hypertension. 2016; 68: e67–e94. doi: 10.1161/HYP.0000000000000053 27977393 10.1161/HYP.0000000000000053 PMC5361411

